# Improvement of refractory migraine headache by propofol: case series

**DOI:** 10.1186/1865-1380-5-19

**Published:** 2012-05-15

**Authors:** Hassan Soleimanpour, Aliakbar Taheraghdam, Rouzbeh Rajaei Ghafouri, Ali Taghizadieh, Karim Marjany, Maryam Soleimanpour

**Affiliations:** 1 Emergency Medicine Department, Tabriz University of Medical Sciences, Daneshgah Street, Tabriz, 51664, Iran; 2 Neurology Department, Tabriz University of Medical Sciences, Daneshgah Street, Tabriz, 51664, Iran; 3 Nikookari Hospital, Tabriz University of Medical Sciences, Daneshgah Street, Tabriz, 51664, Iran; 4 Gastroenterology Research Centre, Tabriz University of Medical Sciences, Daneshgah Street, Tabriz, 51664, Iran

**Keywords:** Propofol, Migraine headache, Visual analogue scale, Emergency department, GABAergic receptors

## Abstract

**Background:**

Several studies have been conducted on managing migraine headaches and developing effective medications for decreasing migraine-associated pain.

**Case presentation:**

Intravenous propofol was prescribed (10 mg every 5 min) for eight patients with intractable migraine headaches visiting the Emergency Department. The average pain score experienced by patients was recorded using the Visual Analogue Scale at the beginning of the treatment procedure and following the injection for 30 min (5-min intervals). The patients’ reported pain scores decreased significantly (*P* = 0.01) from 8.87 ± 0.83 (CI: 8.17, 9.57) to 1.12 ± 0.83 (CI: 0.43, 1.82) before and 30 min following the injection.

**Discussion:**

It seems that in the treatment of intractable migraine headaches, GABAergic receptors, compared to the normal conditions, have a lower activity status.

**Conclusion:**

Because of the high tendency of propofol to GABAergic receptors, it probably changes this physiological condition by activating the receptors, which results in a significant pain reduction.

## Background

The International Association of Headache introduced acetaminophen and NSAIDs as common treatments for mild to moderate migraines, and selective 5-HT agonists (triptans) in more severe cases [1]. However, one of the major problems in the treatment of migraines is refractory migraine headache or migraine pain lasting more than 72 h [2]. Among the various studies that have been conducted worldwide on the treatment of refractory migraine headaches, few have been carried out on the role of propofol in the treatment of such migraine headaches. Propofol (2, 6 di-isopropyl phenol) has been used as a rapid- and short-acting intravenous medication for the induction of anesthesia and procedural sedation for many years. Its pharmacologic mechanism is related to the agonist effect on gamma-aminobutyric acid (GABA) receptors [2]. This study evaluates the role and efficiency of intravenous propofol in several patients presenting to the emergency department.

## Case presentation

In this study, eight patients who presented to the emergency department, Imam Reza Hospital, Tabriz University of Medical Sciences, Tabriz, Iran with headaches were examined [3]. These patients had previously used different medications to alleviate their pain, but had experienced no improvement, and the subjects still complained of severe headaches. At first, the patients’ histories were taken, and complete examinations (general and neurological) were performed. Based on the International Headache Society (IHS) criteria, the subjects were diagnosed with migraines [1]. The migraine headaches in all these eight patients had lasted more than 72 h and had not responded to common treatments (triptans, dexamethasone, opioids and NSAIDs). After diagnosis of migraine headaches for these patients, the manner of using medications, the reason for its prescription and the possible side effects were explained to them, and it was emphasized that propofol is safe and has the fewest side effects. Written consent was obtained from them. Of these eight patients, six were female and two were male, and their average age was 30.5 years old. All patients had experienced migraines for several years. Using a rating scale numbered from 0 to 10 (Visual Analogue Scale, VAS), patients were asked to record their perceived pain on paper (Table 1). In these patients, IV propofol (Propofol-Lipuro 1%, B. Braun) was injected as 10-mg bolus doses every 5 min until achievement of therapeutic response [2]. Severity of the patients’ headaches was measured and recorded every 5 min. The severity of pain based on the VAS on presenting to the ED was reported as 10/10 by two patients, 9/10 by three patients and 8/10 by the other three patients. On this basis, the average severity of pain at presentation was 8.87 ± 0.83 (CI: 8.17, 9.57). Thirty minutes after treatment, the average severity of pain in patients decreased to 1.12 ± 0.83 (CI: 0.43, 1.82) (*P* = 0.01). Before starting treatment, all patients complained of nausea, and five had vomited. Furthermore, four patients complained of photophobia and phonophobia. In all cases, after treatment, concomitant problems such as nausea, vomiting, photophobia and phonophobia had completely resolved. One patient experienced a brief period of drowsiness for 10 min, and in another case, oxygen saturation dropped to 89%, which was appropriately managed by nasal O_2_. Adverse events such as decreased levels of consciousness, changes in blood pressure, tachycardia or bradycardia were not recorded in any of the patients. All patients were followed up by phone for 72 h after release from the ED. Of eight patients being followed up during this time period, six never experienced headache recurrence again and remained without symptoms. However, case 1 experienced a headache and 24 h later rated his pain as 5/10; it was relieved by NSAIDs. Case 8 experienced headache recurrence 36 h later and rated her pain as 4/10. This pain was also appropriately relieved by NSAID_S_.

**Table 1:** Demographic characteristics of patients

**Case**	**Gender**	**Age**	**VAS at presenting**	**VAS at discharge**	**Administered dose of propofol (mg)**	**Required time until palliation of headache (min)**	**Co-existing symptoms**
1	Male	22	10/10	2/10	50	25	Nausea, vomiting, photophobia
2	Female	44	9/10	0/10	10	5	Nausea
3	Female	35	8/10	2/10	20	10	Nausea, vomiting, photo/phonophobia
4	Female	28	10 /10	1/10	20	10	Nausea
5	Female	25	8/10	1/10	30	15	Nausea, vomiting, photo/phonophobia
6	Male	28	8/10	0/10	30	15	Nausea
7	Female	32	9/10	1/10	20	10	Nausea, vomiting
8	Female	30	9/10	2/10	60	30	Nausea, vomiting, photo/phonophobia

In the mentioned cases (1 and 8) there was no need for re-admission to the ED and receiving IV medications since the severity of the pain was less than at the first presentation to the ED (VAS = 10/10 and 9/10).

## Discussion

So far, little work has been done regarding the role of propofol in the treatment of migraine headaches. Common medications used for the treatment of this kind of headache include opioids, NSAIDs, ergotamine compounds, triptans, antiemetics and other analgesics. During acute migraine attacks because of absorption disorders (even in the absence of nausea and vomiting), the intravenous route is preferred [2].

Prescription of intravenous propofol in subanesthetic doses in these eight patients caused pain relief for refractory migraine headaches. Propofol is a medication that is often used for inducing anesthesia in the operating room [3-6]. The effect of propofol on GABA receptors has been well proved by evidence from different studies [2]. Propofol inhibits afferent sympathetic action and cardiac baroreceptor reflexes [7,8]. Another study demonstrated that the effect of this medication on EEG during sedation with low doses is limited to the frontal lobe of the brain [9].

Also, another study indicated a global decrease in metabolism of CNS [10]. Its pharmacological mechanism is due to an agonist effect on gamma-aminobutyric acid (GABA) receptors [2].

It seems that the therapeutic effects of propofol are due to its effects on the chloride channels in the β1-subunit of GABA receptors [11].

Drummond et al. also reported the successful treatment of two cases of resistant migraine headache with intravenous propofol [12].

Krusz et al. reported the first study of propofol use for headaches in 2000. This study indicated that 82 percent of patients were completely pain free. In the rest of them the pain decreased between 50 to 90 percent. None of the subjects experienced any drowsiness or disorientation after propofol prescription. Only a few patients had transient lethargy and unintelligible language (4–3 min), and one other (case 8) had spontaneous finger movements. The researchers concluded that propofol can be used in the clinic or emergency department as a definitive therapy for treatment-resistant migraines without side effects. In this study, the dramatic effect of propofol on pain relief in refractory migraine headaches was mentioned to be due to the high affinity of propofol to GABA receptors, which have a low activity status in migraines. Propofol, by stimulating these receptors, overcomes this physiological process and, as a result, migraine headaches can be cured. Researchers also considered using other medications with the same mechanism (GABA stimulators) as potential medications in treating migraine headaches and other types of headaches; this requires more research and investigation [2].

Furthermore, there are several other reports demonstrating the efficacy and good therapeutic effects of intravenous propofol in the treatment of refractory migraines [13].

As compared with other similar studies, because of its quite large sample size the present study is of significant importance for the assessment of intravenous propofol’s efficacy in the treatment of intractable migraine headaches (case series).

Also, there are studies that investigated the role of propofol in the treatment of non-migraine-type chronic headaches. For example, in a study that was conducted on patients with daily chronic headaches resistant to common treatments, more than 90% of patients showed significant improvements in their headaches, and also no symptoms have been reported. Based on the results of this study, propofol administration under reliable monitoring and in controlled conditions was suggested for daily chronic headaches [14].

High efficiency of intravenous propofol in the treatment of acute resistant migraine headaches can pave the way for new treatments in the field of migraine headaches and also be an appropriate replacement for other common medications for the treatment of migraines. With more complementary research, it will be possible to introduce this medication as a new and quite effective medication in the treatment of acute migraine headaches because of its wonderful effect of decreasing pain, rapid onset of action, safety and few side effects. However, there are still many questions about the pathophysiology and exact mechanism of migraines, and more studies are required to explore this field.

## Conclusion

It seems that in refractory migraine headaches, GABAergic receptors are in a lower activity status [2]. Because of the high tendency of propofol to these receptors, it probably changes this physiological condition by activating the receptors, which results in a significant pain reduction. Therefore, propofol can be introduced as a medication that is effective, rapid-acting, safe and with few side effects for relieving acute migraine headaches.

## Consent

Every aspect of the present study was explained to the patients, and we obtained their written consent for participation in the study.

## Abbreviations

VAS, Visual Analogue Pain Scale; NSAIDs, Nonsteroidal anti-inflammatory drugs.

## Competing interests

The authors declare that they have no competing interests.

## Authors’ contributions

HS, AAT, AT and RG collected clinical data, reviewed the literature on the topic and drafted the manuscript. KM and MS analyzed and interpreted the patient data. All of the authors were involved in patient management or the writing of the manuscript. All authors read and approved the final manuscript.

## Authors’ information

HS is Associate professor of Anesthesiology and Critical Care, Fellowship in Trauma Critical Care and CPR at the Department of Emergency Medicine, Tabriz University of Medical Sciences, Tabriz, Iran. He is also editorial board member of Emergency medicine journal (EGM) and Pakistan Journal of Biological Sciences (PJBS). AAT is Assistant professor of Neurology at the Department of Neurology, Tabriz University of Medical Sciences, Tabriz, Iran. AT and RG are Assistant professor of Internal medicine and Emergency Medicine at the Department of Emergency Medicine, Tabriz University of Medical Sciences, Tabriz, Iran, respectively. KM is an Anesthesiologist in the Nikookari Hospital, Tabriz University of Medical Sciences, Tabriz, Iran. MS is member of Gastroenterology Research Centre, Tabriz University of Medical Sciences, Tabriz, Iran.
